# An integrative systems approach identifies novel candidates in Marfan syndrome‐related pathophysiology

**DOI:** 10.1111/jcmm.14137

**Published:** 2019-01-24

**Authors:** Raghu Bhushan, Lukas Altinbas, Marten Jäger, Marcin Zaradzki, Daniel Lehmann, Bernd Timmermann, Nicholas P. Clayton, Yunxiang Zhu, Klaus Kallenbach, Georgios Kararigas, Peter N. Robinson

**Affiliations:** ^1^ Charité University Hospital Berlin Germany; ^2^ Yenepoya Research Centre Yenepoya (Deemed to be University) Deralakatte Mangalore India; ^3^ Berlin Institute of Health (BIH) Core Genomics Facility Charité, University Medical Center Berlin Germany; ^4^ Department of Cardiac Surgery University Hospital Heidelberg Heidelberg Germany; ^5^ Max Planck Institute for Molecular Genetics Berlin Germany; ^6^ Sanofi Genzyme Framingham Massachusetts; ^7^ Department of Cardiac Surgery INCCI HaerzZenter Luxembourg Luxembourg; ^8^ DZHK (German Centre for Cardiovascular Research) Berlin Germany; ^9^ The Jackson Laboratory for Genomic Medicine Farmington Connecticut

**Keywords:** Chemokine signalling, Igfbp2 signalling, Marfan syndrome, Mfap4, mgR/mgR, RNA‐sequencing, Spp1, TGF‐beta signalling, Transcriptomics

## Abstract

Marfan syndrome (MFS) is an autosomal dominant genetic disorder caused by mutations in the *FBN1* gene. Although many peripheral tissues are affected, aortic complications, such as dilation, dissection and rupture, are the leading causes of MFS‐related mortality. Aberrant TGF‐beta signalling plays a major role in the pathophysiology of MFS. However, the contributing mechanisms are still poorly understood. Here, we aimed at identifying novel aorta‐specific pathways involved in the pathophysiology of MFS. For this purpose, we employed the *Fbn1* under‐expressing mgR/mgR mouse model of MFS. We performed RNA‐sequencing of aortic tissues of 9‐week‐old mgR/mgR mice compared with wild‐type (WT) mice. With a false discovery rate <5%, our analysis revealed 248 genes to be differentially regulated including 20 genes previously unrelated with MFS‐related pathology. Among these, we identified *Igfbp2*,* Ccl8*,* Spp1*,* Mylk2*,* Mfap4*,* Dsp* and *H19*. We confirmed the expression of regulated genes by quantitative real‐time PCR. Pathway classification revealed transcript signatures involved in chemokine signalling, cardiac muscle contraction, dilated and hypertrophic cardiomyopathy. Furthermore, our immunoblot analysis of aortic tissues revealed altered regulation of pSmad2 signalling, Perk1/2, Igfbp2, Mfap4, Ccl8 and Mylk2 protein levels in mgR/mgR vs WT mice. Together, our integrative systems approach identified several novel factors associated with MFS‐aortic‐specific pathophysiology that might offer potential novel therapeutic targets for MFS.

## 1 INTRODUCTION

Marfan syndrome (MFS) is an inherited autosomal connective tissue disorder caused by mutations in an extracellular matrix protein, fibrillin‐1 (*FBN1*), with manifestations in the cardiovascular system, eye, skeleton, lung, skin and dura.^1^ The major complication is aortic dissection, which generally occurs in the presence of aortic root dilatation. In MFS, the extracellular matrix undergoes remodelling with increase in aortic stiffness and progressive weakening of the aortic wall leading to aortic dissection.

Extensive research has been conducted using mouse models with mutant fibrillin‐1 (*Fbn1*) that develop MFS‐like aortopathy despite normal aortic elastogenesis.^2^, ^3^, ^4^ Two mice models, Fbn1^C1039G/+^ and mgR/mgR with different degrees of MFS pathologies, have frequently been used in MFS studies.^3^, ^4^, ^5^, ^6^ In the Fbn1^C1039G/+^ model, it has been observed that 90% of animals develop the variable severe phenotype and ~5% of animals die by 8 months of age as a result of aortic aneurysms.^5^ On contrary, the mgR/mgR (hypomorphic) model produces ~20% normal fibrillin1 that develops a severe form of MFS with an earlier onset and an average survival of 2.5 months.^2^, ^7^

Previous research has suggested that the pathophysiology of MFS can be explained by ongoing abnormalities in TGF‐beta signalling that lead to progressive destabilization of the aortic wall and it has been suggested that fibrillin‐1 interacts with latent TGF‐beta binding proteins (LTBPs) to restrict TGF‐beta activation.^8^, ^9^ Hence, the deficiency of fibrillin‐1 in MFS promotes the release and activation of TGF‐beta. Several studies have shown that there is an increased abundance of TGF‐beta in MFS.^8^, ^10^ In addition, non‐canonical TGF‐beta signalling pathway (extracellular signal‐regulated kinase [ERK] 1 and 2) has also been shown to promote aortic aneurysms in Marfan mice.^11^ TGF‐beta neutralizing antibodies (1D11) and Losartan (an angiotensin II type 1 receptor [AT1] antagonist) have been shown to decrease TGF‐beta signalling in Marfan mouse models. Furthermore, aortic extracts containing elastin/fibrillin fragments with GxxPG motifs significantly increased macrophage chemotaxis.^12^, ^13^ Subsequent treatment of Marfan mice with a neutralizing antibody (BA4 ‐ anti‐elastin, monoclonal antibody) decreased macrophage chemotaxis, inflammation and degeneration of the vessel architecture.^12^

Overall, in the last decade, the leading hypothesis has been that TGF‐beta induction is a major driver of Marfan disease progression. This assumption was recently challenged by selectively deleting the TGF‐type‐II receptor in MFS mice (Fbn1^C1039G/+^) suggesting that increased TGF‐beta in MFS may not be the sole cause of MFS aortopathy.^14^ Consequently, these results suggest that there may be previously unrecognized mechanisms, by which MFS disease progression is regulated and potential etiologies of MFS need further examination.

With an aim to gain insight into the molecular mechanisms and novel transcripts governing the progression of disease in MFS, we performed RNA‐sequencing of aortic tissues from Marfan (mgR/mgR) vs WT mice and identified several new factors associated with Marfan‐induced aortic disease progression.

## 2 MATERIALS AND METHODS

### 2.1 Animal experiments and isolation of ascending aortas from mice

Heterozygous *Fbn1* underexpressing mice (25) were mated to generate homozygous mgR/mgR and WT mice. Genotyping was performed as previously described.^15^ Mice were maintained under pathogen‐free conditions, and the ethical guidelines for animal work and the animal experiments were approved by the Landesamt für Gesundheit und Soziales in Berlin (LaGeSo Reg. No. 0024/14). Mice were sacrificed; the ascending aortas and arch of the mgR/mgR homozygous and WT animals were isolated and pooled for subsequent RNA expression analysis.

### 2.2 Van Giesson staining

Aortic architecture was assessed using elastic van Giesson staining kit from Sigma‐Aldrich, following the manufacturer's instructions.

### 2.3 RNA‐sequencing analysis

Total RNA was extracted from WT and mgR/mgR homozygous animals (9 weeks old animals) using the RNeasy mini kit (Qiagen). Library preparation was done using the TruSeq RNA Library Preparation Kit v2 (Illumina) following the manufacturer's instructions. An Illumina HiSeq 2500 sequencing device was used to perform the high throughput sequencing, to generate 65‐75 million reads of 50‐bp paired‐end reads, with a mean insert size of 150 bp. The reads were mapped to Mus musculus genome build (mm9) using TopHat2.^16^ DESeq2^17^ was used for differential expression analysis with a false discovery rate of 0.05% (FDR).

### 2.4 qRT‐PCRs

Freshly dissected aortic tissues were snap‐frozen in liquid nitrogen. Frozen tissues were pulverized and RNA was isolated using Qiagen RNeasy Mini kit following the manufacturer's instructions. For reverse transcription, 1 microgram of RNA was used for conversion into cDNA using the RevertAid H minus cDNA Synthesis Kit (ThermoFisher Scientific). Real‐time PCR was performed on the Applied Biosystems 7900HT real‐time PCR machine as described previously.^18^ Gapdh was used as endogenous control. Refer to Data S1 for qPCR primers.

### 2.5 Western blots

Aortic samples were homogenized using FastPrep^®^‐24 Classic instrument for 20 seconds. Protein isolation was done using the QIAGEN AllPrep DNA/RNA/Protein Mini Kit, following the manufacturer's instructions, in the presence of protease and phosphatase inhibitors. Proteins were quantified using the Pierce BCA kit. Proteins (30 μg) were loaded on 10% SDS‐PAGE gels and transferred onto nitrocellulose membranes. After blocking (3% milk in 0.1% PBS‐T), membranes were incubated with corresponding primary antibodies overnight at 4°C. Immunoreactive proteins were detected using ECL Plus (GE Healthcare, Buckinghamshire, UK). Refer to Data S1 for antibody details.

### 2.6 Pathway classification

The gene functional annotation was performed using the DAVID functional annotation tool^19^ (https://david.ncifcrf.gov/) followed by KEGG pathway analysis with an ease score of 0.03 and gene count of 5 genes per pathway.

## 3 RESULTS

### 3.1 Differential gene regulation in the aorta of WT vs mgR/mgR mice

**Figure 1:**
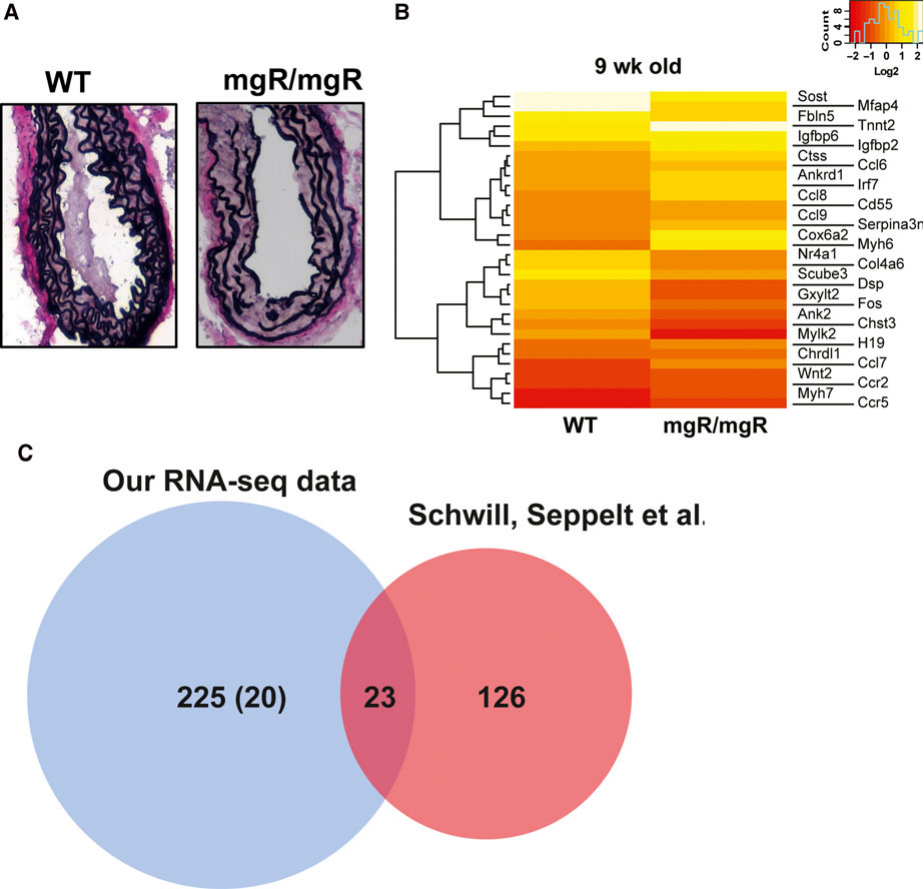
RNA‐sequencing analysis of WT vs mgR/mgR aorta. A, Verhoeff‐van Gieson staining in wild‐type (WT) vs mgR/mgR mouse. Van Gieson staining was performed on aortas isolated from wild‐type and mgR mice. Increased elastin fragmentation and breaks were observed in Marfan mice, compared to wild‐type. B, Differential expression of genes by RNA‐ sequencing analysis in WT vs mgR/mgR aortas (9 wk) animals. Heatmap depicting the Log2 FKPM values of significantly regulated genes (*P* < 0.05). The red colour indicates the low expression of genes and white indicates the high expression of genes. C, Comparison of our RNA‐sequencing data to published microarray (Schwill, Seppelt et al. 2013). Our comparison identified, 23 genes to be commonly regulated between both and 225 genes to be exclusively regulated in our dataset and 126 genes exclusive to Schwill, Seppelt dataset

To identify novel factors and to gain insight into the molecular mechanisms underlying MFS, we employed mgR/mgR mice comparing them with their WT littermates. First, we performed Van Giesson staining to assess the elastic architecture of the aortic tissue and we found the elastic fibres to be fragmented in the mgR/mgR mice compared to WT mice (Figure 1A), a phenomenon commonly seen in MFS.^2^, ^20^

Next, we performed RNA‐sequencing of aortic tissues from homozygous mgR/mgR vs WT mice. Our analysis revealed that 248 genes were differentially regulated between the two groups (Figure 1B, Table S1), with 144 genes being up‐regulated and 104 being down‐regulated in WT vs mgR/mgR mice (Table S1). Importantly, our analysis revealed 20 transcripts previously unknown to be differentially expressed in the aorta of MFS mice (Table S2).

Gene ontology (GO) analysis of the RNA‐sequencing data revealed the enrichment of gene signatures in GO terms, such as adrenergic signalling in cardiomyocytes, dilated and hypertrophic cardiomyopathy (HCM), chemokine signalling, cardiac muscle contraction and calcium signalling pathway, that are relevant to cardiovascular complications and MFS (Table 2, Table S3).

We then compared our RNA‐sequencing dataset (9 weeks old aortas, mgR/mgR mice) to a published microarray dataset (6 weeks old aortas, mgR/mgR mice) 15 and identified 23 genes to be commonly regulated between the two datasets (Figure 1C, Table S4). Interestingly, 225 genes were exclusively regulated in our dataset (Table S5), while 126 genes were exclusively regulated in the previously published microarray dataset^15^ (Table S6). Furthermore, we compared our RNA‐sequencing dataset to other aneurysms such as angiotensin II infused ‐ apolipoprotein deficient mouse model of abdominal aortic aneurysms (AAA, 17 weeks old aortas)^21^ and found 36 genes to be common regulated across both datasets (Table S7, Figure S3).

### 3.2 Validation of selected transcripts by qPCR and Western blotting

**Figure 2:**
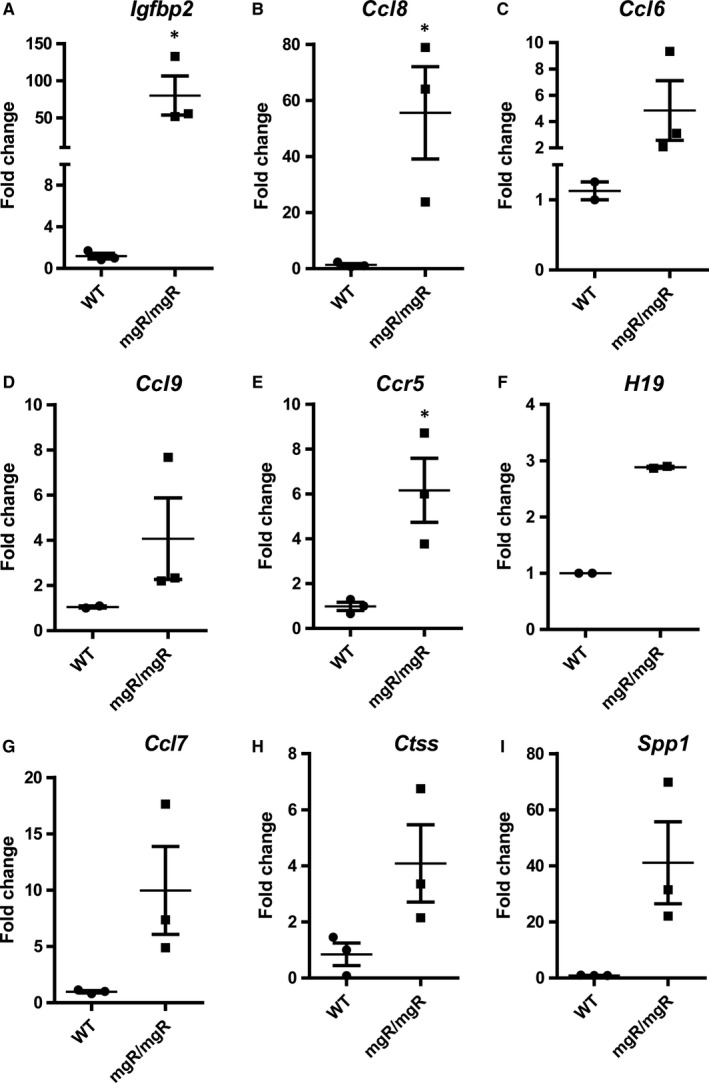
Validation of up‐regulated genes from RNA‐sequencing data. A‐E, qPCRs were performed between WT vs mgR/mgR aortas for the up‐regulated genes from RNA‐sequencing data. Fold changes were calculated and Gapdh was used as normalizing control. Data were validated across n = 3 different biological replicates. Each replicate is derived from three pooled WT vs mgR tissues (11 weeks old)

**Figure 3:**
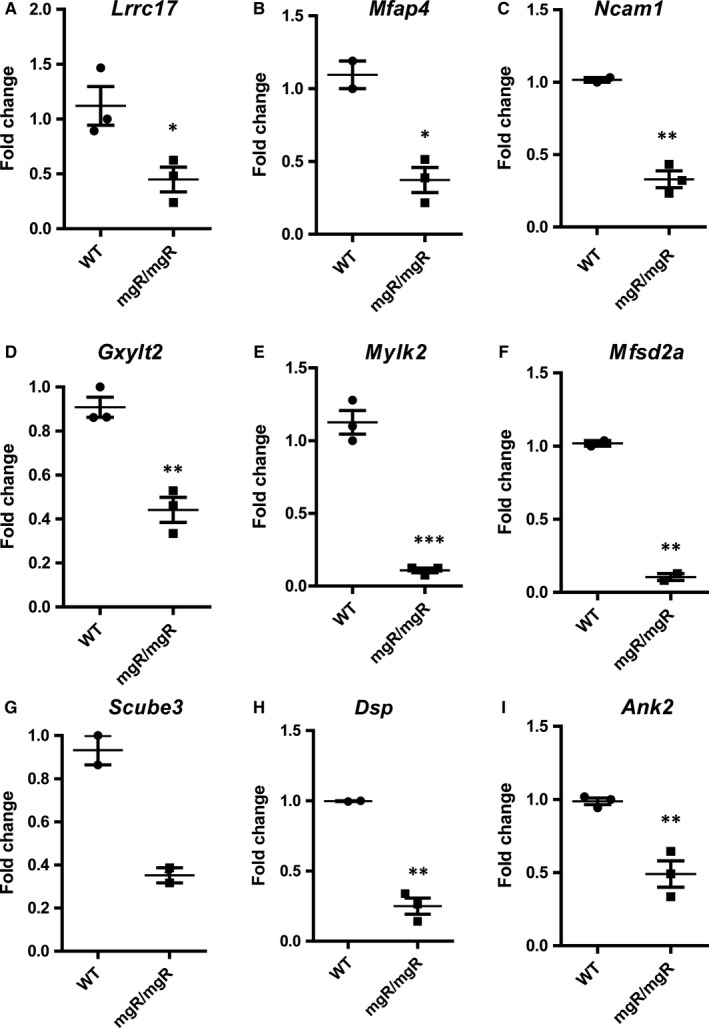
Validation of down‐regulated genes from RNA‐sequencing data. A‐G, qPCR was performed between WT vs mgR/mgR aortas for the down‐regulated genes from RNA‐ sequencing data. Fold changes were calculated and Gapdh was used as a normalizing control. Data were validated across n = 3 or n = 2 different biological replicates. Each replicate is derived from three pooled WT vs mgR tissues (11 weeks old)

**Figure 4:**
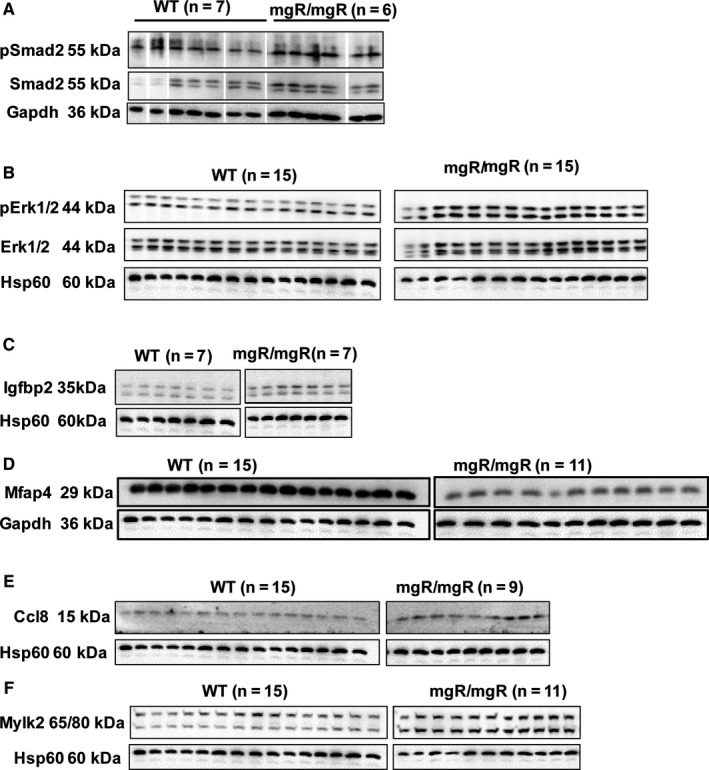
Protein validations of identified genes from RNA‐sequencing data by Western blots. A) pSmad2 and B) pErk1/2 protein levels were induced in mgR/mgR aortic tissues compared to WT (11 weeks old animals). Up‐regulation of C) Igfbp2, E) Ccl8 and F) Mylk2 protein levels and Down‐regulation of D) Mfap4 protein levels in mgR/mgR aortic tissues compared to WT. Protein lysates were harvested as described in methods sections. Gapdh or Hsp60 was used as a loading control

We selected and validated the genes randomly (*Lrrc17*,* Gxylt2*,* Mfsd2a*,* Scube* and *Ank2*) and based on their role in aneurysms (*Mmp12*,* Spp1*,* Ctss*) or cardiovascular complications (*Mfap4*,* Igfbp2*) or inflammatory signalling pathways (*Ccls* and *Ccrs*). Our analysis revealed that many genes enriched in chemokine signalling (*Ccl8*,* Ccr5*,* Ccl9*,* Ccl6*,* Ccl7*,* Ccl2* and *Ccl5)* were highly induced in mgR/mgR aortic tissues (Figure 2B,D‐G and Figure S1). Another novel candidate gene identified from our sequencing analysis, *Igfbp2*, was found to be highly up‐regulated across independent biological replicates, suggesting that this might be a potential factor for MFS (Figure 2A). Other highly up‐regulated candidates of interest were *Spp1*,* Ctss*,* Cd55*,* Cd53*,* Lmod2*,* Irf7*,* Chrdl1*,* Tmem176b*,* Lgals3*,* MMP12* and *H19* LncRNA in mgR/mgR compared to WT mice (Figure 2C,H,I and Figure S1). In addition, we also identified and validated a set of down‐regulated genes by qPCR, such as *Lrrc17*,* Mfap4*,* Ncam1*,* Gxylt2*,* Mfsd2a*,* Scube3*,* Ank2*,* Dsp*,* Myh10*,* Xirp*,* Smoc1*,* Chsy3*,* Adamts17*,* ppp1R36*,* Mylk2*,* Gucy1b3* and *Abcc9* (Figure 3 and Figure S2). The most significantly up‐ and down‐regulated genes from our RNA‐sequencing data are provided as Table 1. We further assessed the protein levels of key genes identified by RNA‐sequencing. pSMAD2 and pErk1/2 signalling pathways were induced in mgR/mgR compared to WT mice (Figure 4A,B). Furthermore, we demonstrated that Igfbp2 and Ccl8 protein levels are induced (Figure 4C,E) and Mfap4 protein levels are decreased in mgR/mgR compared to WT mice (Figure 4D), which correlates with our aortic RNA‐sequencing data. However, we found Mylk2 protein levels to be increased (Figure 4F), contrary to decreased mRNA levels in mgR/mgR compared to WT mice (Figure 3E).

**Table 1:** Genes significantly up‐ or down‐regulated in mgR/mgR aortic aneurysms both in RNA‐sequencing and qPCR datasets

Gene name	RNA‐seq and qPCR (in mgR/mgR)	*P*‐value
*Igfbp2*	Up	5.00E‐05
*Mfap4*	Down	5.00E‐05
*Tmem176b*	Up	0.00065
*Irf7*	Up	5.00E‐05
*Ccl8*	Up	5.00E‐05
*Ccl6*	Up	0.00025
*H19*	Up	2.00E‐04
*Ccl9*	Up	0.00015
*Spp1*	Up	0.00045
*Ctss*	Up	5.00E‐05
*Myh10*	Down	0.00035
*Lrrc17*	Down	5.00E‐05
*Scube3*	Down	5.00E‐05
*Gxylt2*	Down	5.00E‐05
*Ncam1*	Down	5.00E‐05
*Mfsd2a*	Down	0.00015

## 4 DISCUSSION

In this study, we set out to identify novel differentially expressed genes during MFS in mgR/mgR aortas using RNA‐sequencing. Our analysis revealed many previously unknown genes to be significantly regulated in mgR/mgR mice.

The predominant paradigm of MFS pathophysiology in recent years has focussed on alterations of TGF‐beta signalling. Moreover, high levels of circulating TGF‐beta were observed in Marfan mouse models and patients, suggesting it is an important factor involved in MFS.^22^, ^23^ Most of the treatment regimens adopted have been to antagonize TGF‐beta signalling with neutralizing antibodies or Losartan. However, more recent literature demonstrated that the deletion of the TGF‐beta receptor II (Tgfbr2) increases the aortopathy in Marfan animals, thus suggesting a complicated role of TGF‐beta signalling in MFS^7^, ^14^ and also the possibility of other potential regulators and mechanisms underlying the MFS phenotype.

To this extent, one of our novel findings is the regulation of Igfbp2, which was significantly induced in mgR/mgR vs WT aortas in our RNA‐ sequencing, qPCR and immunoblotting data. Igfbp2, a secreted protein, belongs to a class of insulin like growth factor binding proteins (Igfbp1‐6), which bind to IGF1, IGF2 and insulin and signal via IGF1R, IGF‐IIR/M6P and insulin receptors, thus transducing the signal to the nucleus, followed by transcriptional activation of specific genes to promote proliferation and differentiation. There have been conflicting reports about the role of Igfbp2 in cancers, as there is evidence supporting it to be both a tumour suppressor and an oncogene in different cancers.^24^ In glioma cells, Igfbp2 promotes pERK1/2 phosphorylation via integrin β1, thus regulating cell proliferation and invasion.^25^ It is known that pERK1/2 levels are induced in MFS mice and it has been proposed that abolishing pERK1/2 levels could be a potential therapy towards MFS.^11^, ^20^

In addition, it was shown that ERK1/2 signalling is limited to non‐myocyte compartment of MFS mouse heart and upon pressure overload, it contributes to cardiomyopathy, emphasizing ERK signalling to be a crucial therapeutic target in MFS.^26^ Here, we speculate that the induced Igfbp2 levels in mgR/mgR mice could be crucial in regulating pERK1/2 levels in MFS and this needs further investigation.

Furthermore, Mfap4 was highly down‐regulated in mgR/mgR mice. Mfap4 is an extracellular glycoprotein that specifically binds tropoelastin, fibrillin‐1 and fibrillin‐2.^27^ It is expressed in elastic tissues, such as aorta, skin and lung, and is localized to elastic fibres of these tissues.^28^, ^29^, ^30^ In dermal fibroblasts, MFAP4 interacts directly with fibrillin‐1 and plays a crucial role in microfibril development and maintenance of ECM proteins.^31^ In mgR/mgR hypomorphic animals, Fbn1 levels are low (~20% normal Fbn1 produced) and this corresponds with low level of expression of Mfap4 in our data (RNA‐sequencing, qPCR and Western), suggesting a possible important mechanistic role of Mfap4‐fibrillin‐1 interaction in MFS. However, MFAP4 protein levels were found to be up‐regulated in Marfan patients,^32^ suggesting there could be differential regulation of MFAP4 in different disease model systems or between species. Our results on Mfap4 shed light on a potentially crucial regulator in MFS that needs further investigation.

We identified different chemokines to be highly up‐regulated in MFS (Figure 2D‐G). Medial necrosis and presence of inflammatory cells is well known in MFS. T cells (CD8+ and CD4+) have been shown in adventitia of MFS aortas, emphasizing a crucial role of immune cells in both medial and adventitial layers of MFS aorta.^33^ Previously, studies have reported inflammatory genes to be involved in both mouse and human AAA.^15^, ^21^, ^34^ Among the chemokines identified in our data, Ccl8 was highly up‐regulated at both RNA (in RNA‐ sequencing, qPCRs datasets) and protein levels. Apart from *Ccl8*, we identified *Ccr5*,* Ccl5*,* Ccl2*,* Ccl7* and *Ccl9*, to be highly up‐regulated in mgR/mgR mice. *Ccl8* was also shown to be induced during AAA in mice.^21^ The role of IL6‐STAT3 signalling in contributing to aneurysm dilation has been shown in mgR/mgR Marfan model.^35^ In MFS patients, inflammation correlated with aortic disease, emphasizing the crucial role of inflammatory signalling in aortic disease progression.^33^ It is known that defective fibrillin‐1 in MFS leads to impaired collagen and network organization. This might lead to further collagen damage and the release of degradation products that could possibly activate inflammatory pathways.^33^ However, in Fbn1^C1039G/+^ Marfan model the anti‐inflammatory therapy did not show beneficial effect on aortic dilatation, suggesting a complex role of inflammation in different MFS model systems.^36^ Overall, it is unclear, if inflammation is the cause or the consequence of aortic dilation and its role in MFS is not clearly understood. Collectively, our study reveals the importance of several inflammatory genes that can be potential targets for novel anti‐inflammatory therapeutics.

We also found *Spp1*,* Mmp12* and *Ctss* to be significantly up‐regulated in mgR/mgR mice. Spp1 is a multifunctional phosphoprotein, expressed in various cell types and a known biomarker for cardiovascular diseases.^6^, ^37^ Elevated Spp1 levels have been observed in circulation in different cancers, autoimmune disease and AAA. Spp1 has been shown to be involved in the regulation of chronic inflammation and recruitment of T cell and macrophages and regulates cytokine production in macrophages and T cells.^38^ The levels of OPN have been found to be elevated in AAA patients (both in plasma and aortic wall) and it may drive AAA formation.^39^ In addition, the local application of osteopontin enhanced vascular healing by decreasing the calcification and maintaining the luminal integrity in rabbit thoracic aortas.^40^ In addition, a single SNP in TgfbrII had altered Spp1 secretion after adjusting for multiple testing.^41^ However, the mechanistic regulation of Spp1 in Marfan aortic aneurysms is not clear. It is known that the complex pathogenesis of MFS involves an imbalance in expression of matrix metalloproteinases (MMPs) and tissue inhibitors of MMP activity.^42^ More specifically, *Mmp2*,* Mmp9* and *Mmp12* have been shown to be up‐regulated in mgR/mgR mice.^43^ We identified the same trend in *Mmp12* expression. These results emphasize the importance of *Spp1*,* Ctss* and *Mmp12* in mgR/mgR disease progression.

Apart from coding genes, we also found the *H19* LncRNA to be significantly up‐regulated in mgR/mgR mice. H19 is a conserved and imprinted lncRNA having many biological functions in different diseases. Increased levels of *H19* are associated with increased risk of coronary artery disease in patients.^44^ In addition, *H19* was induced in heart failure patients.^45^ *H19* in association with miR‐675 axis was shown to regulate cardiomyocyte apoptosis and diabetic cardiomyopathy.^46^, ^47^ The same axis (H19‐miR‐675) is reported to function as a negative regulator of cardiomyocyte hypertrophy in mice.^48^ However, the role of H19 in context of MFS is unclear and we believe it could be an interesting target to investigate.

Moreover, we identified several genes to be commonly regulated between our RNA‐ sequencing dataset and a previously published microarray datasets,^15^, ^21^ suggesting the significance and importance of these genes in MFS. Furthermore, our approach identified several genes, such as *Lrrc17*,* Mfap4*,* Ncam1*,* Gxylt2*,* Mfsd2a*,* Scube3*,* Ank2*,* Dsp*,* Myh10*,* Xirp*,* Smoc1*,* Chsy3*,* Adamts17*,* ppp1R36*,* Mylk2*,* Gucy1b3* and *Abcc9 to be* significantly down‐regulated in mgR/mgR aortas, whose regulation has not been previously related to MFS. However, we found Mylk2 protein levels to be induced in mgR/mgR mice, suggesting possible posttranscriptional regulation of this particular gene.

GO analysis of differentially regulated genes revealed relevant pathways involved in cardiovascular biology and MFS, such as adrenergic signalling in cardiomyocytes, dilated cardiomyopathy, HCM, calcium signalling pathway, chemokine signalling pathway and others (Table 2). The crucial role of adrenergic signalling in cardiac diseases is recognized. It is known that hyperactivity of β‐adrenergic signalling and increase in ROS production contribute to cardiac hypertrophy and heart failure.^49^, ^50^ In addition, β‐adrenergic blockade using propranolol decreased the aortic dilatation and complications and improved the clinical manifestations of MFS,^51^, ^52^ emphasizing its crucial role in MFS. The other GO enriched pathway from our data was dilated cardiomyopathy that has been shown to be the primary manifestation of MFS, which results from ECM‐induced abnormal mechanosignalling by cardiomyocytes.^53^ Furthermore, dilated cardiomyopathy in an MFS patient was accompanied by chronic type A aortic dissection and right atrial thrombus.^54^ We also found calcium signalling pathway to be overrepresented in our dataset. It is known that calcium influx regulates FBN1 expression.^55^ Moreover, calcium channel blockers, are used as a second line of therapy in 10%‐20% MFS patients who are intolerant to β‐blockers.^56^ However, calcium channel blockers have detrimental effects in MFS mice and are associated with an increased risk of cardiovascular pathology in human MFS.^57^ All these data, highlight the importance of our finding on calcium signalling and its implication in MFS management. Apart from the above mentioned pathways, our data also emphasized the importance of chemokine signalling, which is crucial during MFS.^33^ However, the exact role of chemokine signalling in MFS is still unclear. The other pathways from our GO analysis, such as focal adhesion, HCM and cardiac muscle contraction have also been implicated in MFS.^58^, ^59^, ^60^ Overall, our GO analysis suggests a specific and crucial role of identified genes and pathways that are relevant in mgR/mgR mice.

**Table 2:** KEGG pathway analysis of differentially regulated genes from RNA‐sequencing data

Term	*P*‐value	Genes
Adrenergic signalling in cardiomyocytes	0.00026	*Tnnt2*,* Actc1*,* Adcy7*,* Pln*,* Scn4b*,* Myh7*,* Myh6*,* Tnni3*,* Scn5a*
Dilated cardiomyopathy	0.00042	*Tnnt2*,* Actc1*,* Adcy7*,* Pln*,* Mybpc3*,* Ttn*,* Tnni3*
Focal adhesion	0.00072	*Myl7*,* Lamc3*,* Mylk3*,* Mylk4*,* Mylk2*,* Mylpf*,* Actn2*,* Mapk10*,* Col4a6*,* Spp1*
Cardiac muscle contraction	0.00210	*Tnnt2*,* Actc1*,* Cox6a2*,* Myh7*,* Myh6*,* Tnni3*
cAMP signalling pathway	0.00858	*Fos*,* Htr1b*,* Adcy7*,* Chrm2*,* Gria1*,* Pln*,* Mapk10*,* Tnni3*
Gastric acid secretion	0.01023	*Kcnj15*,* Adcy7*,* Mylk3*,* Mylk4*,* Mylk2*
cGMP‐PKG signalling pathway	0.01252	*Irs2*,* Irs3*,* Adcy7*,* Pln*,* Mylk3*,* Mylk4*,* Mylk2*
Regulation of actin cytoskeleton	0.01284	*Myl7*,* Chrm2*,* Mylk3*,* Mylk4*,* Mylk2*,* Mylpf*,* Actn2*,* Fgf12*
Hypertrophic cardiomyopathy (HCM)	0.01404	*Tnnt2*,* Actc1*,* Mybpc3*,* Ttn*,* Tnni3*
Calcium signalling pathway	0.01955	*Adcy7*,* Tnnc2*,* Chrm2*,* Pln*,* Mylk3*,* Mylk4*,* Mylk2*
Chemokine signalling pathway	0.02828	*Adcy7*,* Ccr5*,* Ccr2*,* Ccl9*,* Ccl8*,* Ccl7*,* Ccl6*
Circadian entrainment	0.02853	*Kcnj5*,* Fos*,* Adcy7*,* Gria1*,* Per1*

In conclusion, our analysis expands the understanding of the pathobiology of Marfan disease progression. Our analysis revealed several genes previously unknown to be involved in aorta‐specific Marfan disease that may be important targets for designing more appropriate therapeutic strategies for the treatment of MFS.

## CONFLICT OF INTEREST

The authors declare no potential conflict of interest.

## References

[1] von Kodolitsch Y , Robinson PN . Marfan syndrome: an update of genetics, medical and surgical management. Heart. 2007;93:755‐760. 17502658 10.1136/hrt.2006.098798 PMC1955191

[2] Pereira L , Lee SY , Gayraud B , et al. Pathogenetic sequence for aneurysm revealed in mice underexpressing fibrillin‐1. Proc Natl Acad Sci USA. 1999;96:3819‐3823. 10097121 10.1073/pnas.96.7.3819 PMC22378

[3] Pereira L , Andrikopoulos K , Tian J , et al. Targetting of the gene encoding fibrillin‐1 recapitulates the vascular aspect of Marfan syndrome. Nat Genet. 1997;17:218‐222. 9326947 10.1038/ng1097-218

[4] Judge DP , Biery NJ , Keene DR , et al. Evidence for a critical contribution of haploinsufficiency in the complex pathogenesis of Marfan syndrome. J Clin Invest. 2004;114:172‐181. 15254584 10.1172/JCI20641 PMC449744

[5] Chung AW , Yang HH , van Breemen C . Imbalanced synthesis of cyclooxygenase‐derived thromboxane A2 and prostacyclin compromises vasomotor function of the thoracic aorta in Marfan syndrome. Br J Pharmacol. 2007;152:305‐312. 17641673 10.1038/sj.bjp.0707391 PMC2042958

[6] Ahmed M , Behera R , Chakraborty G , et al. Osteopontin: a potentially important therapeutic target in cancer. Expert Opin Ther Targets. 2011;15:1113‐1126. 21718227 10.1517/14728222.2011.594438

[7] Cook JR , Clayton NP , Carta L , et al. Dimorphic effects of transforming growth factor‐beta signaling during aortic aneurysm progression in mice suggest a combinatorial therapy for Marfan syndrome. Arterioscler Thromb Vasc Biol. 2015;35:911‐917. 25614286 10.1161/ATVBAHA.114.305150 PMC4376614

[8] Neptune ER , Frischmeyer PA , Arking DE , et al. Dysregulation of TGF‐beta activation contributes to pathogenesis in Marfan syndrome. Nat Genet. 2003;33:407‐411. 12598898 10.1038/ng1116

[9] Kaartinen V , Warburton D . Fibrillin controls TGF‐beta activation. Nat Genet. 2003;33:331‐332. 12610545 10.1038/ng0303-331

[10] Ng CM , Cheng A , Myers LA , et al. TGF‐beta‐dependent pathogenesis of mitral valve prolapse in a mouse model of Marfan syndrome. J Clin Invest. 2004;114:1586‐1592. 15546004 10.1172/JCI22715 PMC529498

[11] Holm TM , Habashi JP , Doyle JJ , et al. Noncanonical TGFbeta signaling contributes to aortic aneurysm progression in Marfan syndrome mice. Science. 2011;332:358‐361. 21493862 10.1126/science.1192149 PMC3111087

[12] Guo G , Munoz‐Garcia B , Ott CE , et al. Antagonism of GxxPG fragments ameliorates manifestations of aortic disease in Marfan syndrome mice. Hum Mol Genet. 2013;22:433‐443. 23100322 10.1093/hmg/dds439

[13] Guo G , Gehle P , Doelken S , et al. Induction of macrophage chemotaxis by aortic extracts from patients with Marfan syndrome is related to elastin binding protein. PLoS ONE. 2011;6:e20138. 21647416 10.1371/journal.pone.0020138 PMC3103536

[14] Wei H , Hu JH , Angelov SN , et al. Aortopathy in a mouse model of Marfan syndrome is not mediated by altered transforming growth factor beta signaling. J Am Heart Assoc. 2017;6:e004968. 28119285 10.1161/JAHA.116.004968 PMC5523644

[15] Schwill S , Seppelt P , Grunhagen J , et al. The fibrillin‐1 hypomorphic mgR/mgR murine model of Marfan syndrome shows severe elastolysis in all segments of the aorta. J Vasc Surg. 2013;57:1628‐1636, 36 e1‐3. 23294503 10.1016/j.jvs.2012.10.007

[16] Kim D , Pertea G , Trapnell C , Pimentel H , Kelley R , Salzberg SL . TopHat2: accurate alignment of transcriptomes in the presence of insertions, deletions and gene fusions. Genome Biol. 2013;14:R36. 23618408 10.1186/gb-2013-14-4-r36 PMC4053844

[17] Love MI , Huber W , Anders S . Moderated estimation of fold change and dispersion for RNA‐seq data with DESeq2. Genome Biol. 2014;15:550. 25516281 10.1186/s13059-014-0550-8 PMC4302049

[18] Ott CE , Grunhagen J , Jager M , et al. MicroRNAs differentially expressed in postnatal aortic development downregulate elastin via 3′ UTR and coding‐sequence binding sites. PLoS ONE. 2011;6:e16250. 21305018 10.1371/journal.pone.0016250 PMC3031556

[19] da Huang W , Sherman BT , Lempicki RA . Systematic and integrative analysis of large gene lists using DAVID bioinformatics resources. Nat Protoc. 2009;4:44‐57. 19131956 10.1038/nprot.2008.211

[20] Habashi JP , Doyle JJ , Holm TM , et al. Angiotensin II type 2 receptor signaling attenuates aortic aneurysm in mice through ERK antagonism. Science. 2011;332:361‐365. 21493863 10.1126/science.1192152 PMC3097422

[21] Rush C , Nyara M , Moxon JV , Trollope A , Cullen B , Golledge J . Whole genome expression analysis within the angiotensin II‐apolipoprotein E deficient mouse model of abdominal aortic aneurysm. BMC Genom. 2009;10:298. 10.1186/1471-2164-10-298 PMC2728106 19580648

[22] Matt P , Schoenhoff F , Habashi J , et al. Circulating transforming growth factor‐beta in Marfan syndrome. Circulation. 2009;120:526‐532. 19635970 10.1161/CIRCULATIONAHA.108.841981 PMC2779568

[23] Franken R , den Hartog AW , de Waard V , et al. Circulating transforming growth factor‐beta as a prognostic biomarker in Marfan syndrome. Int J Cardiol. 2013;168:2441‐2446. 23582687 10.1016/j.ijcard.2013.03.033

[24] Pickard A , McCance DJ . IGF‐binding protein 2 ‐ oncogene or tumor suppressor? Front Endocrinol (Lausanne). 2015;6:25. 25774149 10.3389/fendo.2015.00025 PMC4343188

[25] Han S , Li Z , Master LM , Master ZW , Wu A . Exogenous IGFBP‐2 promotes proliferation, invasion, and chemoresistance to temozolomide in glioma cells via the integrin beta1‐ERK pathway. Br J Cancer. 2014;111:1400‐1409. 25093489 10.1038/bjc.2014.435 PMC4183856

[26] Rouf R , MacFarlane EG , Takimoto E , et al. Nonmyocyte ERK1/2 signaling contributes to load‐induced cardiomyopathy in Marfan mice. JCI Insight. 2017;2:91588. 28768908 10.1172/jci.insight.91588 PMC5543913

[27] Pilecki B , Holm AT , Schlosser A , et al. Characterization of microfibrillar‐associated protein 4 (MFAP4) as a tropoelastin‐ and fibrillin‐binding protein involved in elastic fiber formation. J Biol Chem. 2016;291:1103‐1114. 26601954 10.1074/jbc.M115.681775 PMC4714194

[28] Wulf‐Johansson H , Lock Johansson S , Schlosser A , et al. Localization of microfibrillar‐associated protein 4 (MFAP4) in human tissues: clinical evaluation of serum MFAP4 and its association with various cardiovascular conditions. PLoS ONE. 2013;8:e82243. 24349233 10.1371/journal.pone.0082243 PMC3862580

[29] Toyoshima T , Yamashita K , Furuichi H , Shishibori T , Itano T , Kobayashi R . Ultrastructural distribution of 36‐kD microfibril‐associated glycoprotein (MAGP‐36) in human and bovine tissues. J Histochem Cytochem. 1999;47:1049‐1056. 10424889 10.1177/002215549904700809

[30] Schlosser A , Thomsen T , Shipley JM , et al. Microfibril‐associated protein 4 binds to surfactant protein A (SP‐A) and colocalizes with SP‐A in the extracellular matrix of the lung. Scand J Immunol. 2006;64:104‐116. 16867155 10.1111/j.1365-3083.2006.01778.x

[31] Kasamatsu S , Hachiya A , Fujimura T , et al. Essential role of microfibrillar‐associated protein 4 in human cutaneous homeostasis and in its photoprotection. Sci Rep. 2011;1:164. 22355679 10.1038/srep00164 PMC3240987

[32] Pilop C , Aregger F , Gorman RC , et al. Proteomic analysis in aortic media of patients with Marfan syndrome reveals increased activity of calpain 2 in aortic aneurysms. Circulation. 2009;120:983‐991. 19720936 10.1161/CIRCULATIONAHA.108.843516

[33] Radonic T , de Witte P , Groenink M , et al. Inflammation aggravates disease severity in Marfan syndrome patients. PLoS ONE. 2012;7:e32963. 22479353 10.1371/journal.pone.0032963 PMC3316543

[34] Schonbeck U , Sukhova GK , Gerdes N , Libby P . T(H)2 predominant immune responses prevail in human abdominal aortic aneurysm. Am J Pathol. 2002;161:499‐506. 12163375 10.1016/S0002-9440(10)64206-X PMC1850720

[35] Ju X , Ijaz T , Sun H , et al. IL‐6 regulates extracellular matrix remodeling associated with aortic dilation in a fibrillin‐1 hypomorphic mgR/mgR mouse model of severe Marfan syndrome. J Am Heart Assoc. 2014;3:e000476. 24449804 10.1161/JAHA.113.000476 PMC3959679

[36] Franken R , Hibender S , den Hartog AW , et al. No beneficial effect of general and specific anti‐inflammatory therapies on aortic dilatation in Marfan mice. PLoS ONE. 2014;9:e107221. 25238161 10.1371/journal.pone.0107221 PMC4169510

[37] Waller AH , Sanchez‐Ross M , Kaluski E , Klapholz M . Osteopontin in cardiovascular disease: a potential therapeutic target. Cardiol Rev. 2010;18:125‐131. 20395697 10.1097/CRD.0b013e3181cfb646

[38] Lund SA , Giachelli CM , Scatena M . The role of osteopontin in inflammatory processes. J Cell Commun Signal. 2009;3:311‐322. 19798593 10.1007/s12079-009-0068-0 PMC2778587

[39] Wang SK , Green LA , Gutwein AR , et al. Osteopontin may be a driver of abdominal aortic aneurysm formation. J Vasc Surg. 2018;68:22S‐29S. 29402664 10.1016/j.jvs.2017.10.068 PMC12517559

[40] Seipelt RG , Backer CL , Mavroudis C , et al. Local delivery of osteopontin attenuates vascular remodeling by altering matrix metalloproteinase‐2 in a rabbit model of aortic injury. J Thorac Cardiovasc Surg. 2005;130:355‐362. 16077399 10.1016/j.jtcvs.2004.12.040

[41] Biros E , Clancy P , Norman PE , Golledge J . A genetic polymorphism in transforming growth factor beta receptor‐2 is associated with serum osteopontin. Int J Immunogenet. 2009;36:241‐244. 19601999 10.1111/j.1744-313X.2009.00855.x

[42] Ikonomidis JS , Jones JA , Barbour JR , et al. Expression of matrix metalloproteinases and endogenous inhibitors within ascending aortic aneurysms of patients with Marfan syndrome. Circulation. 2006;114:I365‐I370. 16820601 10.1161/CIRCULATIONAHA.105.000810

[43] Guo G , Ott CE , Grunhagen J , et al. Indomethacin prevents the progression of thoracic aortic aneurysm in Marfan syndrome mice. Aorta (Stamford). 2013;1:5‐12. 26798667 10.12945/j.aorta.2013.13.007 PMC4682693

[44] Zhang Z , Gao W , Long QQ , et al. Increased plasma levels of lncRNA H19 and LIPCAR are associated with increased risk of coronary artery disease in a Chinese population. Sci Rep. 2017;7:7491. 28790415 10.1038/s41598-017-07611-z PMC5548926

[45] Greco S , Zaccagnini G , Perfetti A , et al. Long noncoding RNA dysregulation in ischemic heart failure. J Transl Med. 2016;14:183. 27317124 10.1186/s12967-016-0926-5 PMC4912721

[46] Zhang Y , Zhang M , Xu W , Chen J , Zhou X . The long non‐coding RNA H19 promotes cardiomyocyte apoptosis in dilated cardiomyopathy. Oncotarget. 2017;8:28588‐28594. 28430627 10.18632/oncotarget.15544 PMC5438674

[47] Li X , Wang H , Yao B , Xu W , Chen J , Zhou X . lncRNA H19/miR‐675 axis regulates cardiomyocyte apoptosis by targeting VDAC1 in diabetic cardiomyopathy. Sci Rep. 2016;6:36340. 27796346 10.1038/srep36340 PMC5087087

[48] Liu L , An X , Li Z , et al. The H19 long noncoding RNA is a novel negative regulator of cardiomyocyte hypertrophy. Cardiovasc Res. 2016;111:56‐65. 27084844 10.1093/cvr/cvw078

[49] Madamanchi A . Beta‐adrenergic receptor signaling in cardiac function and heart failure. Mcgill J Med. 2007;10:99‐104. 18523538 PMC2323471

[50] Corbi G , Conti V , Russomanno G , et al. Adrenergic signaling and oxidative stress: a role for sirtuins? Front Physiol. 2013;4:324. 24265619 10.3389/fphys.2013.00324 PMC3820966

[51] Shores J , Berger KR , Murphy EA , Pyeritz RE . Progression of aortic dilatation and the benefit of long‐term beta‐adrenergic blockade in Marfan's syndrome. N Engl J Med. 1994;330:1335‐1341. 8152445 10.1056/NEJM199405123301902

[52] Habashi JP , Judge DP , Holm TM , et al. Losartan, an AT1 antagonist, prevents aortic aneurysm in a mouse model of Marfan syndrome. Science. 2006;312:117‐121. 16601194 10.1126/science.1124287 PMC1482474

[53] Cook JR , Carta L , Benard L , et al. Abnormal muscle mechanosignaling triggers cardiomyopathy in mice with Marfan syndrome. J Clin Invest. 2014;124:1329‐1339. 24531548 10.1172/JCI71059 PMC3934180

[54] Kahveci G , Erkol A , Yilmaz F . Dilated cardiomyopathy in a patient with Marfan syndrome accompanied by chronic type A aortic dissection and right atrial thrombus. Intern Med. 2010;49:2583‐2586. 21139296 10.2169/internalmedicine.49.3880

[55] Benarroch L , Aubart M , Gross MS , et al. Marfan syndrome variability: investigation of the roles of sarcolipin and calcium as potential transregulator of FBN1 expression. Genes (Basel). 2018;9:E421. 30134586 10.3390/genes9090421 PMC6162465

[56] Cook JR , Carta L , Galatioto J , Ramirez F . Cardiovascular manifestations in Marfan syndrome and related diseases; multiple genes causing similar phenotypes. Clin Genet. 2015;87:11‐20. 24867163 10.1111/cge.12436

[57] Doyle JJ , Doyle AJ , Wilson NK , et al. A deleterious gene‐by‐environment interaction imposed by calcium channel blockers in Marfan syndrome. Elife. 2015;4:e08648. 26506064 10.7554/eLife.08648 PMC4621743

[58] Crosas‐Molist E , Meirelles T , Lopez‐Luque J , et al. Vascular smooth muscle cell phenotypic changes in patients with Marfan syndrome. Arterioscler Thromb Vasc Biol. 2015;35:960‐972. 25593132 10.1161/ATVBAHA.114.304412

[59] Owens GK , Kumar MS , Wamhoff BR . Molecular regulation of vascular smooth muscle cell differentiation in development and disease. Physiol Rev. 2004;84:767‐801. 15269336 10.1152/physrev.00041.2003

[60] Teekakirikul P , Eminaga S , Toka O , et al. Cardiac fibrosis in mice with hypertrophic cardiomyopathy is mediated by non‐myocyte proliferation and requires Tgf‐beta. J Clin Invest. 2010;120:3520‐3529. 20811150 10.1172/JCI42028 PMC2947222

