# Supplementary material for: An integrative systems approach identifies novel candidates in Marfan syndrome‐related pathophysiology
Source: J Cell Mol Med. 2019 Jan 24;23(4):2526–35. doi: 10.1111/jcmm.14137 (PMC6433740; doi:10.1111/jcmm.14137)
Supplement: Supplementary file 11 [file JCMM-23-2526-s011.docx]

| **Gene** | **Forward primer** | **Reverse primer** |
| --- | --- | --- |
| Abcc9 | GCAGAAGAAAAAGGCTGCCG | CTCAGCAGGATTGGTCTCCC |
| **Adamts17** | **TTGGCCTCATCCAGCTTGAG** | **GCCCATTTGCGTCTGATCAG** |
| **Ank2** | **TCGCATCAAACAGGACAGCA** | **TACTCGAATGCGCTTGGTGA** |
| Ccl2 | AGTTAACGCCCCACTCACC | CAGCTTCTTTGGGACACCTG |
| Ccl5 | GTGCCCACGTCAAGGAGTAT | CCCACTTCTTCTCTGGGTTG |
| **Ccl6** | **TGCTGCTTCTCTTATGCCACA** | **TTGATGCACCCACCACTGG** |
| Ccl7 | CCCAAGAGGAATCTCAAGAGC | ACTTCCATGCCCTTCTTTGTC |
| Ccl8 | CTGCTCATAGCTGTCCCTGTC | TAGCTTTTCAGCACCCGAAG |
| **Ccl9** | **TGTTTCACATGGGCTTTCAA** | **CCACTGGTGGGAAAATAACC** |
| Ccr5 | GCCATAAAGTCGCTTCTTGC | ACCCCAAGTTCTGCTGACTC |
| **Cd53** | TGTGGCTGTTGCATTTTGGG | GCCAAGTGTCAGGAAGGGAA |
| **Cd55** | **ACATTGTCCAGAGCCACCAA** | **ACCAGGATGAAGCCTTTGTCA** |
| **Chrdl1** | **TGGAACCACTTACCAACATGGA** | **GACTGCACTGATTGGGTTGC** |
| **Chsy3** | **CGATTCCTACCCTCCCCAGA** | **ATGTAGACATCGTCGTCGGC** |
| Ctss | ACATTCAGCTCCCGTTTGGT | AAGAAGGAGGAATGGCTGGC |
| Dsp | AAGCGTCAGGTGCAGAACTT | TTTGTAGTCGCAGAGAGCCC |
| Gucy1b3 | CAGCCCTTACACCTTCTGCA | GGGAGCACTCTGTAGATGGC |
| **Gxylt2** | **GGACTCGCTTCTCTACGTGG** | **CAGCAAGCTGGGTGGAGTTA** |
| H19 | CACTGAAGGCGAGGATGACA | GGATGACTGCCCTTCTGTCC |
| Igfbp2 | TGATGACGACCACTCTGAGG | CTGCTACCACCTCCCAACAT |
| **Irf7** | **CCCAGATGCGTGTTCCTGTA** | **CTGATCTGGGAGCTCAGCAG** |
| **Lmod2** | **AGGAGCTGCTCTTAGGACCA** | **GGGAGATGACCAGGGAGACT** |
| **Lrrc17** | TTGAGAGCGAGGCGTTCTTT | AGGAGCGGCGTGTAAATGAA |
| **Lgals3** | TGCCTTCCACTTTAACCCCC | GGGGAAGGCTGACTGTCTTT |
| Mfap4 | CTGCGACATGACAACTGAGG | CCAGCTTGTAGTCGCTCCAG |
| **Mfsd2a** | CCCCTGGCCATCATTGCTTA | CAGGTACCAAAGGAAGCCGT |
| Mmp12 | GATGAGGCAGAAACGTGGAC | TGACTTTGGATTATTGGAATGC |
| **Myh10** | **GTTATGGTGGAGCTGGCAGA** | **AAGCATGTCAGCTCTGCCAT** |
| Mylk2 | CCCCCTAACGCGAAGAAAGA | ACCGGGTCACCCTTTTTCTC |
| Ncam1 | GACGGCCATGGAACTAGAGG | CTGATGTTCCGGGTGGATGT |
| Ppp1r36 | AGGAAGAGGTAGGCAGGCTT | GCGCTTCTTCTCTCCTCCTG |
| **Scube3** | **TGTGGATGAGTGTGCAGAGG** | **AGGTGTGCTGGTTGTCACTG** |
| **Smoc1** | **ATCGAGGTCGATGCAAAGAT** | **CACTCTGGGACAAACACAGC** |
| Spp1 | TGGTGCCTGACCCATCTCA | TTCATTGGAATTGCTTGGAAGA |
| **Tmem176b** | **TGCATCATGCTCACAGTGGT** | **GGAGCTCCGGCCATACATAC** |
| **Xirp** | **TTCGGTGTTGCCTCTGTCTC** | **TGCCCAATGCTGTCTAAGGG** |

1. **Primer details:**
2. **Antibody details:**

| **Antibody** | **Catalog no** | **Species** | **kDa** | **Primary dilution** |
| --- | --- | --- | --- | --- |
| pSmad2 | 3101S | Rabbit | 55 | 500 |
| Smad2 | 3103S | Mouse | 55 | 1000 |
| Gapdh | AM4300 | Mouse | 36 | 1000 |
| pErk1/2 | 9101S | Rabbit | 44 | 1000 |
| Erk1/2 | 4695P | Rabbit | 44 | 1000 |
| Ccl8 | ab39635 | Rabbit | 11 | 1000 |
| Hsp60 | cs-4870 | Rabbit | 60 | 1000 |
| Mylk2 | ab155506 | Rabbit | 65/80 | 1000 |
| Mfap4 | ab80319 | Rabbit | 29 | 1000 |
